# A145 EFFECT OF DUPILUMAB ON WEIGHT IN PATIENTS AGED 1 TO 11 YEARS WITH ACTIVE EOSINOPHILIC ESOPHAGITIS (EOE): RESULTS FROM THE PHASE 3 EOE KIDS STUDY

**DOI:** 10.1093/jcag/gwae059.145

**Published:** 2025-02-10

**Authors:** M Chehade, R D Pesek, C Menard-Katcher, I Gutmark-Little, N Fugere, R Liu, L Robinson, J Maloney, M Louisias, A Radin

**Affiliations:** Mount Sinai Center for Eosinophilic Disorders, Icahn School of Medicine at Mount Sinai, New York, NY; Division of Allergy/Immunology, Department of Pediatrics, University of Arkansas for Medical Sciences and Arkansas Children’s Hospital, Little Rock, AR; Division of Gastroenterology Hepatology and Nutrition, Department of Pediatrics, University of Colorado School of Medicine, Gastrointestinal Eosinophilic Diseases Program, Digestive Health Institute, Children’s Hospital Colorado, Aurora, CO; Division of Endocrinology, Cincinnati Children’s Hospital Medical Center, Cincinnati, OH; Sanofi, Bridgewater, NJ; Regeneron Pharmaceuticals Inc., Tarrytown, NY; Sanofi, Cambridge, MA; Regeneron Pharmaceuticals Inc., Tarrytown, NY; Sanofi, Cambridge, MA; Regeneron Pharmaceuticals Inc., Tarrytown, NY

## Abstract

**Background:**

During the phase 3 eosinophilic esophagitis (EoE) KIDS study (NCT04394351), dupilumab improved histologic and endoscopic endpoints and quality of life vs placebo in children aged 1 to 11 years with active EoE.

**Aims:**

This analysis assessed the effect of higher-exposure dupilumab vs placebo on weight in children with EoE enrolled in the KIDS study.

**Methods:**

Part A was a 16-week, double-blind treatment period; patients were randomized to receive weight-tiered dupilumab or placebo. Patients who completed Part A were eligible to enter a 36-week, extended treatment period (Part B) in which dupilumab patients continued the same dupilumab regimen and placebo patients switched to pre-assigned dupilumab. Outcomes at Weeks 16 and 52 included mean absolute and percent change from baseline in: body weight-for-age percentile, body mass index (BMI)-for-age z-score for patients aged ≥2 years, weight-for-age z-score, and weight-for-length z-score.

**Results:**

At Week 16, mean percent changes (standard deviation) from Part A baseline for higher-exposure dupilumab vs placebo were +3.09 (+21%) vs +0.29 (+5%) in body weight-for-age percentile; +0.10 (+27%) vs -0.14 (+6%) in BMI-for-age z-score, and +0.12 (-20%) vs -0.01 (-1%) in weight-for-age z-score. At Week 52, improvements were maintained or increased with continued higher-exposure dupilumab; improvements were also observed in placebo patients who switched to higher-exposure dupilumab (**Table**). Dupilumab safety was consistent with its known safety profile.

**Conclusions:**

Higher-exposure dupilumab was associated with a greater increase vs placebo in body weight-for age percentile at Weeks 16 and 52, and sustained trends for increased BMI-for-age and weight-for-age z-scores, in children with EoE aged 1 to 11 years.

**Table. Effect of higher-exposure dupilumab on outcomes associated with weight increases at Weeks 16 and 52**

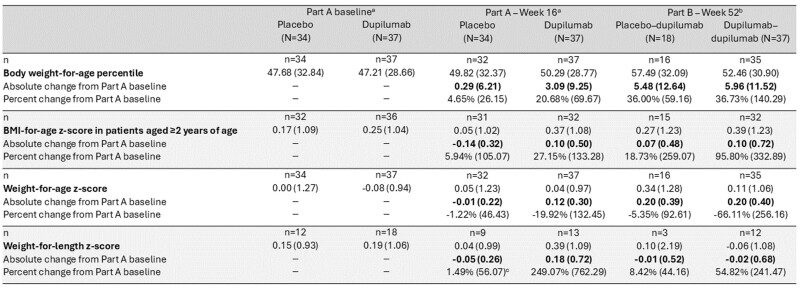

Data are mean (standard deviation). n=number of patients at each visit. ^a^Part A full analysis set. ^b^Part B safety analysis set. ^c^n=8.

BMI, body mass index.

**Funding Agencies:**

Research sponsored by Sanofi and Regeneron Pharmaceuticals Inc.

